# Cytoprotective effect of 2-carbomethoxy-2,3-epoxy-3-prenyl-1,4-naphthoquinone (CMEP-NQ) is mediated by the inhibition of BAK-dependent mitochondrial apoptosis pathway

**DOI:** 10.1371/journal.pone.0204585

**Published:** 2018-10-01

**Authors:** Do Youn Jun, Won Young Jang, Ki Yun Kim, Mi Hee Woo, Young Ho Kim

**Affiliations:** 1 Laboratory of Immunobiology, School of Life Science and Biotechnology, College of Natural Sciences, Kyungpook National University, Daegu, Korea; 2 College of Pharmacology, Daegu Catholic University, Gyeongsan, South Korea; Columbia University, UNITED STATES

## Abstract

The inhibitory mechanism of 2-carbomethoxy-2,3-epoxy-3-prenyl-1,4-naphthoquinone (CMEP-NQ) against apoptosis induced by the microtubule-damaging agents (MDAs), nocodazole (NOC) and 2-methoxyestradiol (2-MeO-E_2_), or a DNA-damaging agent (DDA), camptothecin (CPT) were investigated in human Jurkat T cell clones (J/Neo and J/BCL-XL cells). Treatment of J/Neo cells with NOC, 2-MeO-E_2_, or CPT caused cytotoxicity and apoptotic DNA fragmentation but these events were significantly attenuated in the presence of CMEP-NQ. Although not only MDA (NOC or 2-MeO-E_2_)-induced mitotic arrest, CDK1 activation, and BCL-2, BCL-XL and BIM phosphorylation, but also DDA (CPT)-induced S-phase arrest and ATM-CHK1/CHK2-p53 pathway activation were not or were barely affected in the presence of CMEP-NQ, the levels of anti-apoptotic BAG3 and MCL-1, which were markedly downregulated after MDA- or DDA-treatment, were rather elevated by CMEP-NQ. Under the same conditions, MDA- or DDA-induced mitochondrial apoptotic events including BAK activation, mitochondrial membrane potential (Δψm) loss, caspase-9 activation, and PARP cleavage were significantly inhibited by CMEP-NQ. While MDA- or DDA-induced sub-G_1_ peak and Δψm loss were abrogated in J/BCL-XL cells, MDA-induced mitotic arrest and DDA-induced S-arrest were more apparent in J/BCL-XL cells than in J/Neo cells. Simultaneously, the induced cell cycle arrest in J/BCL-XL cells was not significantly disturbed by CMEP-NQ. MDA- or DDA-treatment caused intracellular reactive oxygen species (ROS) production; however, MDA- or DDA-induced ROS production was almost completely abrogated in J/BCL-XL cells. MDA- or DDA-induced ROS production in J/Neo cells was significantly suppressed by CMEP-NQ, but the suppressive effect was hardly observed in J/BCL-XL cells. Together, these results show that CMEP-NQ efficiently protects Jurkat T cells from apoptotic cell death via the elevation of BAG3 and MCL-1 levels, which results in the inhibition of intrinsic BAK-dependent mitochondrial apoptosis pathway, as does the overexpression of BCL-XL.

## Introduction

Mitochondria, double membrane-bound organelles, are present in most aerobic eukaryotic cells and play a key role in the generation of ATP via electron transport and oxidative phosphorylation. In addition to their role in providing cellular energy, mitochondria are involved in several essential cellular processes, including the regulation of calcium signaling [1], cell cycle control and growth [2], and apoptotic signaling pathways [3]. The importance of mitochondrial function in cells has been well reflected by the finding that mitochondrial dysfunction causes cellular damage and is linked to human diseases and aging [4,5].

Many studies have reported that cells can undergo apoptosis as a response to numerous physiological and nonphysiological signals such as oxidative stress [6], growth factor withdrawal [7,8], corticosteroids [9,10], heat shock [11], irradiation [12], and chemotherapeutic agents [13]. Apoptotic cell death is considered to involve at least two death signaling pathways, namely, the extrinsic death receptor-dependent pathway [14] and the intrinsic mitochondria-dependent pathway [15]. Although the initial triggers provoking these apoptotic induction pathways are different, mitochondrial damage and the release of mitochondrial apoptosis inducers, such as cytochrome *c*, apoptosis-inducing factor (AIF), and reactive oxygen species (ROS), into the cytosol have been frequently associated with apoptosis induction caused by cellular stress and cytotoxic conditions. This clearly indicates a critical involvement of mitochondria in determining cell fate [3,16].

Despite significant advances in our understanding of the molecular mechanisms underlying mitochondrial disorders that lead to unwanted cell injury and organ failure, effective treatments targeting mitochondria have not yet been developed. Therefore, mitochondria-targeted approaches that prevent mitochondrial damage and improve mitochondrial function have been proposed as promising strategies for the prevention and amelioration of mitochondrial dysfunction-associated cellular damage and human diseases.

A naturally produced naphthoquinone, 2-carbomethoxy-2,3-epoxy-3-prenyl-1,4-naphthoquinone (CMEPNQ, C_17_H_16_O_5_, MW 300.3), was initially identified and purified from the roots of *Rubia cordifolia* L., which have been used in Asian traditional medicine for the treatment of arthritis, kidney stones, inflammation of the joints, hemostasis, uteritis, and psoriasis [17,18]. Recently, we reported that CMEP-NQ inhibits the progression of 3T3-L1 preadipocytes into mature adipocytes through two different inhibitory mechanisms. First, it induces apoptotic cell death when dosed at a high concentration (40 μM), and second, it suppresses adipocytic differentiation without exerting cytotoxicity when dosed at a low concentration (10 μM) [19]. More recently, we have shown that CMEP-NQ (3.5–14.0 μM) suppresses the lipopolysaccharide (LPS)-induced production of nitric oxide (NO), prostaglandin E_2_, and pro-inflammatory cytokines (IL-1β, IL-6, and TNF-α) in a RAW264.7 murine macrophage cell line [20]. The anti-inflammatory effect of CMEP-NQ is exerted by inhibition of TLR4-mediated MyD88-dependent events, including the association of MyD88 with IRAK1 and subsequent activation of NF-κB and AP-1 and the generation of ROS, as well as by the inhibition of TLR4-mediated TRIF-dependent activation of IRF3 and subsequent induction of iNOS expression. Although CMEP-NQ does not possess in vitro free-radical scavenging activity, which is easily detected by a well-known antioxidant N-acetylcysteine (NAC), it blocks ROS production in LPS-stimulated RAW264.7 cells more efficiently than NAC.

As numerous studies have reported that excess ROS levels cause mitochondrial deterioration leading to apoptosis induction [21–24], we sought to examine whether CMEP-NQ can block induced apoptosis in human Jurkat T cells treated with either microtubule-damaging agents (MDAs) or DNA-damaging agents (DDAs), in which intrinsic mitochondrial damage and ROS elevation are involved. To investigate the protective mechanisms of CMEP-NQ against MDA- or DDA-induced mitochondrial damage and intracellular ROS production, we evaluated the effect of CMEP-NQ on the induced intrinsic BAK-dependent apoptotic events. This was carried out by using one of two MDAs [nocodazole (NOC) and 2-methoxyestradiol (2-MeO-E_2_)] or a DDA [camptothecin (CPT)] and human acute leukemia Jurkat T cell clones stably transfected with an empty vector (J/Neo) or the *BCL-XL* expression vector (J/BCL-XL) that causes the overexpression of anti-apoptotic BCL-XL [25]. The results show that CMEP-NQ prevents mitochondrial damage via the blockade of BAK activation and caspase cascade activation through the upregulation of anti-apoptotic BCL-2-associated athanogene 3 (BAG3) and myeloid cell leukemia 1 (MCL-1) levels, which protects the cells from apoptotic cell death induced by MDA or DDA treatment. Additional results show that CMEP-NQ abrogates MDA- or DDA-induced ROS production, which occurs as a consequence of mitochondrial damage in J/Neo cells undergoing apoptosis.

## Materials and methods

### Reagents, antibodies, and cells

CPT, 2-MeO-E_2_, NOC, 3,3’-dihexyloxacarbocyanine iodide (DiOC_6_), and 4’,6-diamidino-2-phenylindole (DAPI) were purchased from Sigma Chemical (St. Louis, MO, USA). 2-Carbomethoxy-2,3-epoxy-3-prenyl-1,4-naphthoquinone (CMEP-NQ, C_17_H16O_5_, MW 300.3), used in this study was purified in our previous study [20]. An ECL western blot kit was purchased from Amersham (Arlington Heights, IL, USA), and the Immunobilon-P membrane was obtained from Millipore Corporation (Bedford, MA, USA). Anti-poly (ADP-ribose) polymerase (PARP), anti-BAK, anti-BIM, anti-BAG3, anti-BCL-2, anti-MCL-1, anti-CDK1, anti-cyclin B1, and anti-p53 antibodies were purchased from Santa Cruz Biotechnology (Santa Cruz, CA, USA). Ant-caspase-9, anti-p-CDK1 (Tyr-15), anti-p-CDK1 (Thr-161), anti-p-BCL-2 (Ser-70), anti-p-MCL-1 (Ser-159/Thr-163), anti-p-p53 (Ser-15), anti-p53-upregulated modulator of apoptosis (PUMA), anti-ATM, anti-p-ATM (Ser-1981), anti-CHK1, anti-p-CHK1 (Ser-317), anti-CHK2, and anti-p-CHK2 (Ser-19) antibodies were purchased from Cell Signaling Technology (Beverly, MA, USA). Anti-BAK (Ab-1) antibody was purchased from Calbiochem (San Diego, CA, USA). The ROS sensitive probe dihydroethidium (DHE) was purchased from Santa Cruz Biotechnology. Human acute leukemia Jurkat T cell clones, stably transfected with a *BCL-XL* expression vector (J/BCL-XL) or with an empty vector (J/Neo) were kindly provided by Dr. Dennis Taub (Gerontology Research Center, NIA/NIH, Baltimore, MD, USA). Both J/Neo cells and J/BCL-XL cells were maintained in RPMI 1640 medium containing 10% fetal bovine serum (FBS), 20 mM HEPES (pH 7.0), 100 μM β-mercaptoethanol, 100 μg/ml gentamicin, and 400 μg/ml G418 (A.G. Scientific Inc., San Diego, CA, USA). J/BCL-XL cells overexpressing BCL-XL and J/Neo cells were identified using western blot analysis. These stable clones were maintained in culture for no more than 3 months before the studies, and were used in a number of our previous investigations including recent studies [26,27].

### Cytotoxicity assay and DNA fragmentation analysis

The cytotoxic effect of NOC, 2-MeO-E2, or CPT on J/Neo and J/BCL-XL cells was analyzed using a trypan blue exclusion test of cell viability [28]. Cells (5 × 10^5^ cells/mL) were pretreated with 7.5 μM CMEP-NQ for 1 h prior to incubation with 0.3 μM NOC, 1.0 μM 2-MeO-E_2_, or 0.02 μM CPT. At 17 h after incubation, the cell suspension was mixed with an equal volume of 0.4% trypan blue, and the mixture was allowed to incubate for approximately 2 min at room temperature prior to counting in a hemocytomter.

Equivalent cultures were prepared and cells were collected to analyze apoptotic DNA fragmentation using a Triton X-100 lysis method with 1.2% agarose gel electrophoresis as previously described [26].

### Flow cytometric analysis

Flow cytometric analysis was used to measure the cell cycle state of Jurkat T cells exposed to NOC, 2-MeO-E_2_, or CPT in the absence or presence of CMEP-NQ and was performed using a FACS Calibur (BD Science, San Jose, CA, USA) as described elsewhere [26]. The changes in the mitochondrial membrane potential (Δ*ψ*m) following treatment with 0.3 μM NOC, 1.0 μM 2-MeO-E_2_, or 0.02 μM CPT in the absence or presence of CMEP-NQ were measured after staining with DiOC_6_ [27,29]. Activation of BAK in Jurkat T cells following the same treatments was measured as described previously [30]. To measure intracellular ROS level, the cells were treated with DHE at 37°C for 30 min, and the mean fluorescent intensity (MFI) was analyzed using flow cytometry (FACS Aria III system, BD Science, USA) at an excitation wavelength of 488 nm.

### Preparation of cell lysates and western blot analysis

Cell lysates were prepared by suspending 5 × 10^6^ Jurkat T cells in 300 μL of lysis buffer, as described elsewhere. An equivalent amount of protein lysate (20 μg) was electrophoresed on a 4–12% NuPAGE gradient gel (Invitrogen/Novex, Carlsbad, CA, USA) with MOPS buffer and then was electrotransferred to an Immobilon-P membrane. Protein detection was performed using an ECL western blot kit according to the manufacturer’s instructions. Densitometry was performed using ImageQuant TL software (Amersham). The arbitrary densitometric units for each protein of interest were normalized to the densitometric units for GAPDH.

### Statistical analysis

Unless otherwise indicated, each result in this study is a representative of at least three separate experiments. Values are expressed as the means ± standard deviation (SD) of these experiments. Statistical significance was calculated using Student′s *t*-test. *P* values <0.05 were considered significant.

## Results

### Inhibitory effect of CMEP-NQ on NOC-, 2-MeO-E_2_-, and CPT-induced apoptotic DNA fragmentation, apoptotic sub-G_1_, and Δψm loss in Jurkat T cells

**Fig 1:**
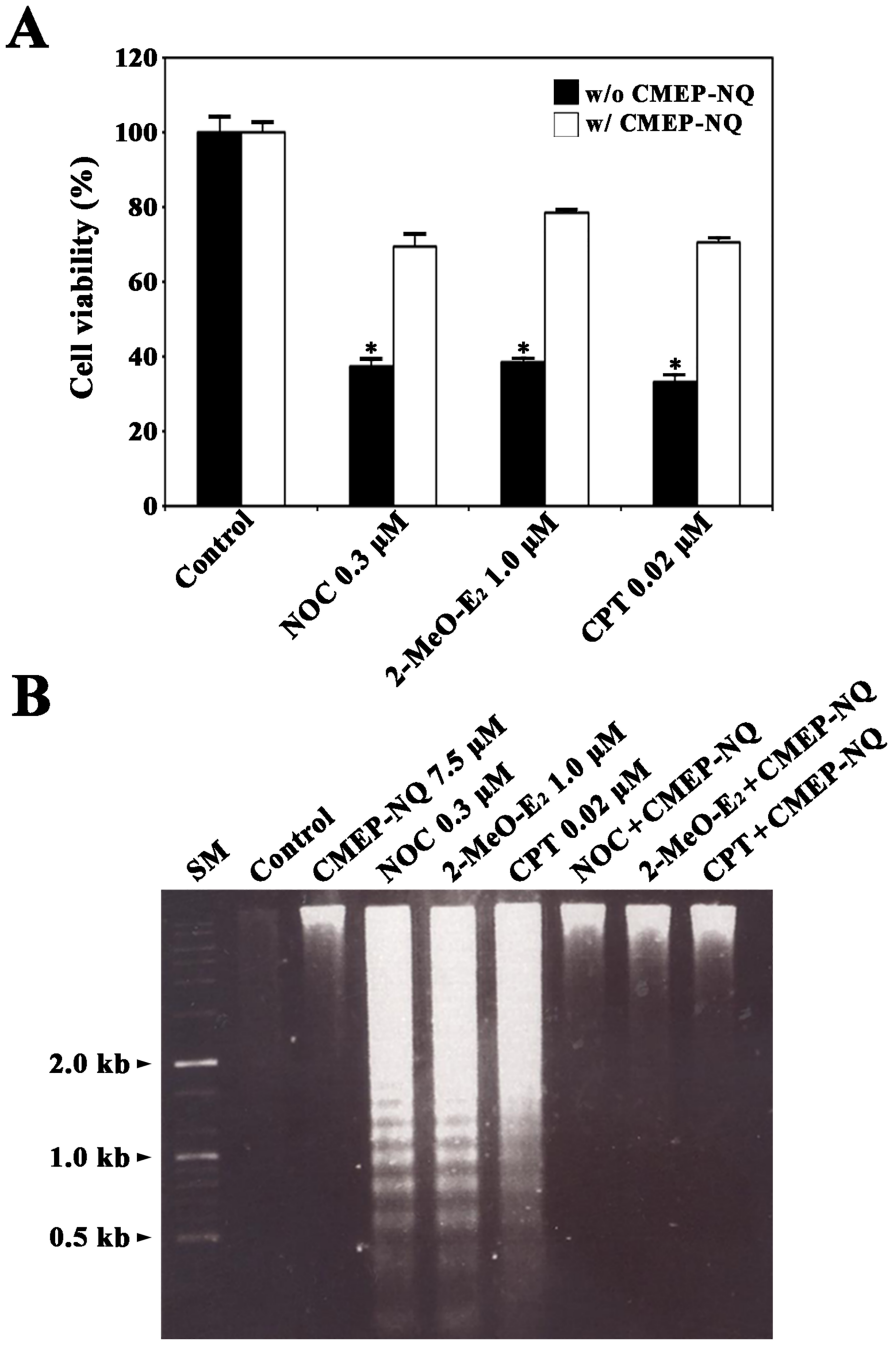
Cytoprotective effect of CMEP-NQ on NOC-, 2-MeO-E_2_-, and CPT-induced cytotoxicity (A) and apoptotic DNA fragmentation (B) in the human Jurkat T cell clone J/Neo cells. The cells were exposed to 7.5 μM CMEP-NQ for 1 h prior to treatment with 0.3 μM NOC, 1.0 μM 2-MeO-E_2_, or 0.02 μM CPT for 17 h. Cell viability was measured using a trypan blue exclusion test. Each value is expressed as the mean ± SEM (n = 3; three replicates per independent experiment). *P < 0.05 compared with the control. Equivalent cultures were prepared and processed for apoptotic DNA fragmentation analysis by the Triton X-100 lysis method using 1.2% agarose gel electrophoresis. A representative result is shown; two additional experiments yielded similar results.

To examine whether CMEP-NQ protects cells from mitochondrial damage and apoptotic cell death, we evaluated the effect of CMEP-NQ on the cytotoxicity and apoptotic DNA fragmentation induced by NOC, 2-MeO-E_2_, and CPT in J/Neo cells, which were previously reported to occur through mitochondria-dependent caspase cascade activation [31–33]. The results of the trypan blue exclusion assay showed that the viabilities of J/Neo cells treated with 0.3 μM NOC, 1.0 μM 2-MeO-E_2_, and 0.02 μM CPT for 17 h were 37.6%, 38.6%, and 33.4%, respectively, whereas concomitant treatment with 7.5 μM CMEP-NQ markedly increased the respective cellular viabilities to 69.8%, 78.3%, and 70.4% (Fig 1A). Moreover, the apoptotic DNA fragmentation was induced in J/Neo cells after treatment with 0.3 μM NOC, 0.1 μM 2-MeO-E_2_, or 0.02 μM CPT but not in the concomitant presence of 7.5 μM CMEP-NQ, indicating of protective effect of CMEP-NQ on MDA- and DDA-induced apoptosis (Fig 1B).

**Fig 2:**
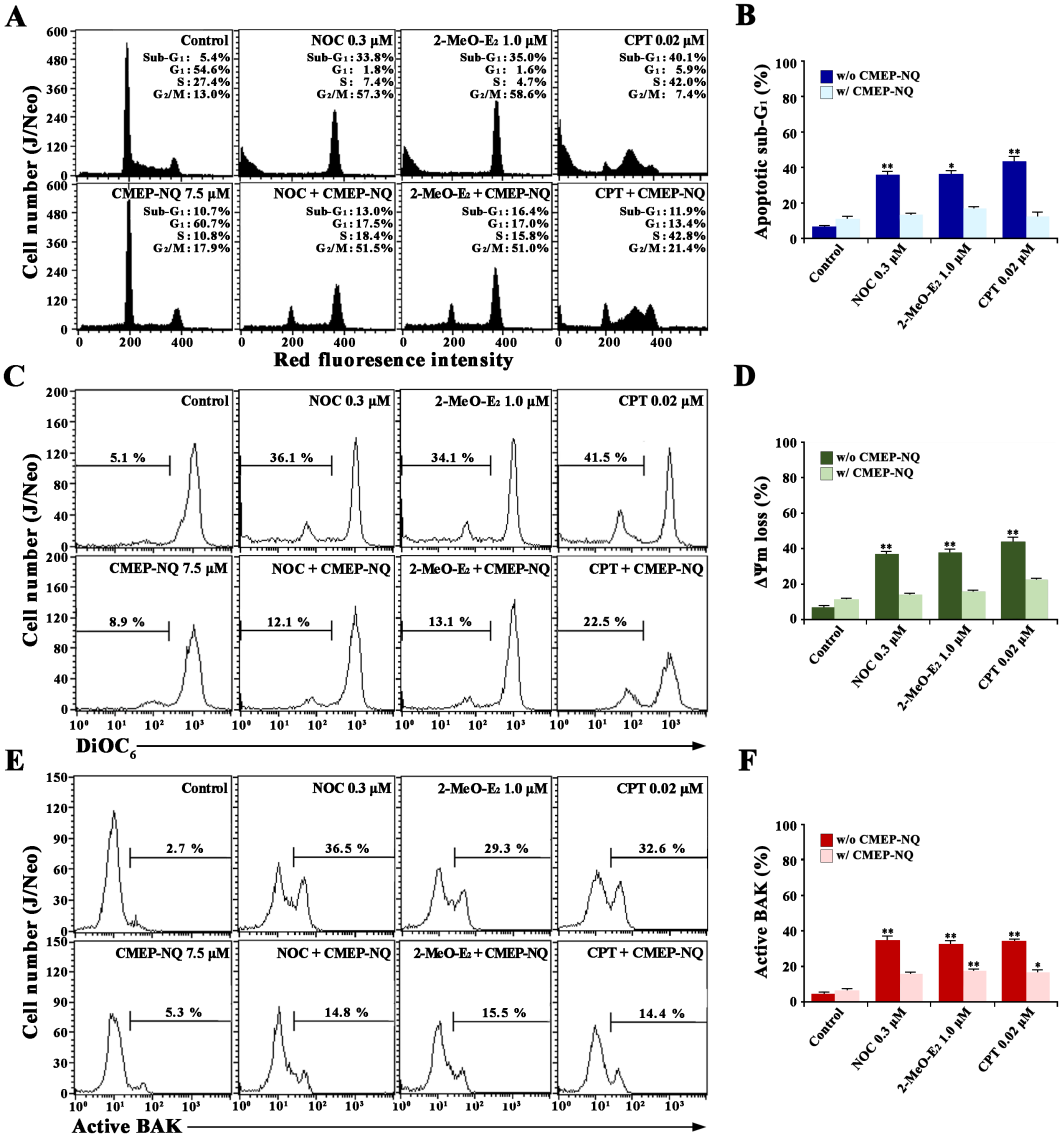
Inhibitory effect of CMEP-NQ on NOC-, 2-MeO-E_2_-, and CPT-induced alterations in cell cycle distribution (A and B), Δψm loss (C and D), and BAK activation (E and F) in J/Neo cells. After pretreatment with 7.5 μM CMEP-NQ for 1 h, J/Neo cells (5 × 10^5^/mL) were treated with 0.3 μM NOC, 1.0 μM 2-MeO-E_2_, or 0.02 μM CPT for 17 h and then subjected to flow cytometric analysis of cell cycle distribution, Δψm loss, and BAK activation as described in the Materials and Methods. A representative result is shown; two additional experiments yielded similar results. Error bars represent standard deviations with * and ** indicating *P <* 0.05 and *P <* 0.01, respectively, compared with the control.

Previously, it has been shown that the apoptogenic effect of NOC and 2-MeO-E_2_ depends on M phase cell cycle arrest and that of CPT depends on S phase cell cycle arrest prior to the induction of apoptosis [31–33]. To examine whether CMEP-NQ affected cell cycle progression, we investigated the effect of CMEP-NQ on NOC-, 2-MeO-E_2_-, or CPT-induced apoptotic perturbation of the cell cycle distribution in J/Neo cells. As shown in Fig 2A and 2B, cells treated with NOC and 2-MeO-E_2_ showed 33.8% and 35.0% apoptotic sub-G_1_ phase cells, 1.8% and 1.6% G_1_ phase cells, 7.4% and 4.7% S phase cells, and 57.3% and 58.6% G_2_/M phase cells, respectively. In contrast, the proportions of NOC- and 2-MeO-E_2_-induced apoptotic sub-G_1_ phase cells decreased to 13.0% and 16.4% with 17.5% and 17.0% G_1_ phase cells, 18.4% and 15.8% S phase cells, and 51.5% and 51.0% G_2_/M phase cells, respectively, in the presence of 7.5 μM CMEP-NQ. Additionally, cells treated with CPT showed 40.1% apoptotic sub-G_1_ cells, 5.9% G_1_ cells, 42.0% S cells, and 7.4% G_2_/M cells, respectively, whereas the rates of apoptotic sub-G_1_, G_1_, S, and G_2_/M phase cells were 11.9%, 13.4%, 42.8%, and 21.4%, respectively, when treated with CPT in the presence of 7.5 μM CMEP-NQ. These results indicate that, although the effect of CMEP-NQ on NOC-, 2-MeO-E_2_-, or CPT-induced cell cycle disturbance is not remarkable, the apoptogenic activities of MDAs (NOC or 2-MeO-E_2_) and DDA (CPT) were significantly inhibited by 53–70% in the presence of CMEP-NQ. These results also suggest that the anti-apoptotic activity of CMEP-NQ is mediated by a direct inhibition of induced apoptosis and not by cell cycle disturbance.

To examine whether the inhibitory effect of CMEP-NQ on NOC-, 2-MeO-E_2_, or CPT-induced apoptotic DNA fragmentation and apoptotic sub-G_1_ peak is due to its capability of preventing mitochondrial damage that leads to Δ*ψ*m loss, we compared changes in the Δ*ψ*m of J/Neo cells treated with NOC, 2-MeO-E_2_, or CPT alone with those of J/Neo cells treated concomitantly with CMEP-NQ using flow cytometry with DiOC_6_ staining. As shown in Fig 2C and 2D, although J/Neo cells, exponentially growing, showed negligible Δ*ψ*m loss, the cells treated with 0.3 μM NOC, 1.0 μM 2-MeO-E_2_ or 0.02 μM CPT for 17 h showed 36.1%, 34.1%, and 41.5% Δ*ψ*m loss, respectively. However, the levels of NOC-, 2-MeO-E_2_-, and CPT-induced Δ*ψ*m loss in J/Neo cells decreased to 13.0%, 16.4%, and 11.9%, respectively, with concomitant 7.5 μM CMEP-NQ treatment, indicating a suppressive effect of CMEP-NQ on MDA- and DDA-induced Δψm loss.

To further examine the specific apoptotic process that is inhibited by CMEP-NQ, we used flow cytometry with conformation-specific anti-BAK (Ab-1) antibody [30] to analyze whether induced BAK activation caused by NOC, 2-MeO-E_2_, or CPT was affected by CMEP-NQ treatment. As shown in Fig 2E and 2F, BAK activation was not detected in exponentially growing J/Neo cells, whereas 36.5%, 29.3%, and 32.6% of the cells treated with NOC, 2-MeO-E_2_, and CPT showed BAK activation, respectively; however, the levels of induced BAK activation were reduced to 14.8%, 15.5%, and 14.4%, respectively, after concomitant treatment with CMEP-NQ. These results show that BAK activation, which is responsible for Δ*ψ*m loss, is targeted by the anti-apoptotic action of CMEP-NQ.

### Effect of CMEP-NQ on NOC-, 2-MeO-E_2_- and CPT-induced apoptotic signaling pathways leading to BAK activation

**Fig 3:**
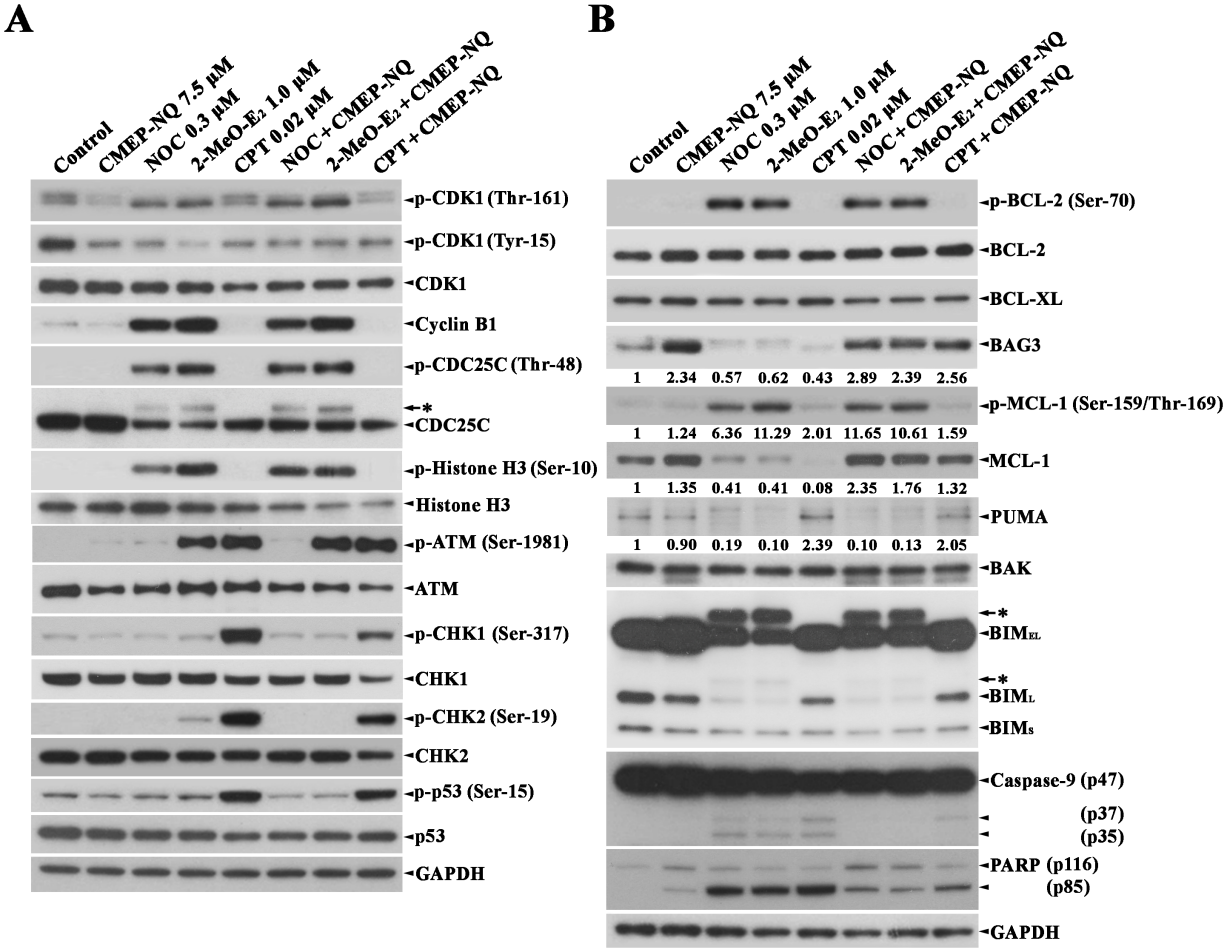
Western blot analysis of phosphorylated CDK1 (Thr-161), phosphorylated CDK1 (Tyr-15), CDK1, cyclin B1, phosphorylated-CDC25C (Thr-48), CDC25C, phosphorylated-Histone H3 (Ser-10), Histone H3, phosphorylated-CHK2 (Ser-19), CHK2, phosphorylated p53 (Ser-15), p53, and GAPDH (A), and phosphorylated BCL-2 (Ser-70), BCL-2, BCL-XL, BAG3, phosphorylated MCL-1 (Ser-159/Thr-169), MCL-1, BAK, reduction in the electrophoretic mobility of the BIM isoforms (BIM_EL_ and BIM_L_), caspase-9, PARP, and GAPDH (B) in J/Neo cells treated with NOC, 2-MeO-E_2_, or CPT in the absence and presence of CMEP-NQ for 17 h. After pretreatment with 7.5 μM CMEP-NQ for 1 h, J/Neo cells (5 × 10^5^/mL) were treated with 0.3 μM NOC, 1.0 μM 2-MeO-E2, or 0.02 μM CPT for 17 h and then collected for the preparation of total cell lysates. The levels of individual proteins in the cell lysates were examined by western blotting as described in the Materials and Methods. Symbol: ←*, the phosphorylated form of CDC25C and BIM. A representative result is shown; two additional experiments yielded similar results.

As BAK-mediated Δ*ψ*m loss is one of the initial intracellular changes associated with mitochondria-dependent apoptosis [16], our results suggest that CMEP-NQ may be a promising agent to alleviate unwanted mitochondrial damage and the resultant mitochondrial apoptosis. To examine the molecular mechanism underlying CMEP-NQ-mediated inhibition of BAK activation in J/Neo cells treated with NOC, 2-MeO-E_2_, or CPT, we investigated the effect of CMEP-NQ on the proximal apoptotic events crucial for BAK activation by western blot analysis. When J/Neo cells were mitotically arrested by treatment with NOC or 2-MeO-E_2_, the level of inhibitory phosphorylation of CDK1 at Tyr-15 decreased, the level of activating phosphorylation of CDK1 at Thr-161 increased, and the expression of cyclin B1 increased (Fig 3A). Moreover, histone H3 phosphorylation at Ser-10 by aurora kinase 2, which is regulated by CDK1 during the G_2_/M phase [34,35], increased. CDC25C phosphorylation at Thr-48, which is necessary for CDK1 dephosphorylation at Tyr-15, also increased. The cellular changes associated with mitotic CDK1 activation, which was induced by NOC- or 2-MeO-E_2_-treatment, were not affected by CMEP-NQ. BCL-2 phosphorylation at Ser-70, MCL-1 phosphorylation at Ser-159 and/or Thr-163, and BIM (BIM_EL_ and BIM_L_) phosphorylation, as evidenced by their phosphorylation-induced reduction in mobility during SDS-polyacrylamide gel electrophoresis, increased in J/Neo cells treated with NOC or 2-MeO-E_2_ (Fig 3B). BCL-2, BCL-XL, and BAK expression levels remained relatively constant after treatment with NOC or 2-MeO-E_2_, whereas the MCL-1 expression level markedly decreased with a significant increase in its phosphorylation level. Although the phosphorylation levels of BCL-2, MCL-1, and BIM were not affected by CMEP-NQ, the levels of MCL-1, not including the BCL-2 and BIM levels, which remained constant, increased in the presence of CMEP-NQ. Interestingly, anti-apoptotic BAG3 levels were significantly reduced by NOC or 2-MeO-E_2_ but remarkably upregulated by concomitant treatment with CMEP-NQ. In accordance with the CMEP-NQ-mediated upregulation of BAG3 and MCL-1 levels in J/Neo cells treated with NOC or 2-MeO-E_2_, the caspase-9 activation that proceeded through proteolytic cleavage of the inactive proenzyme (47 kDa) to its active forms (37/35 kDa) and PARP cleavage were not or were hardly detected in the presence of CMEP-NQ. These results indicate that CDK1 is activated, and its enzymatic activity is sustained and involved in BAK activation during NOC- and 2-MeO-E_2_-induced mitotic arrest. Moreover, the inhibitory effect of CMEP-NQ on NOC- and 2-MeO-E_2_-induced BAK activation is not associated with the CDK1 activation pathway, which contributes to BAK activation via phosphorylation of BCL-2, MCL-1, and BIM. Additionally, these results raise the possibility that the CMEP-NQ-mediated upregulation of MCL-1 and BAG3 levels was responsible for its inhibitory effect on NOC- and 2-MeO-E_2_-induced BAK activation.

To examine how CMEP-NQ inhibits CPT-induced BAK activation, we decided to investigate CPT-mediated apoptotic events upstream of BAK activation, such as the phosphorylation of p53 at Ser-15 and CHK2 phosphorylation at Ser-19 [31], by western blot analysis. The results of the western blot analysis showed that the activating phosphorylation of ATM, CHK1, CHK2, and p53 increased significantly in J/Neo cells after 0.02 μM CPT treatment; however, these CPT-induced activating phosphorylations were not affected by CMEP-NQ. To further examine the inhibitory effect of CMEP-NQ on CPT-induced apoptotic events, which might play critical roles in BAK activation, any changes in anti-apoptotic and pro-apoptotic protein levels were investigated. The protein levels of BCL-2, BCL-XL, and BAK remained relatively constant in CPT-treated J/Neo cells, whereas the BAG3 and MCL-1 levels decreased by 0.43-fold and 0.08-fold, respectively, and the PUMA level was elevated by 2.39-fold, compared to those values in untreated J/Neo cells. However, J/Neo cells treated with CMEP-NQ alone showed 2.34- and 1.35-fold increases in BAG3 and MCL-1 levels, respectively. Concomitant treatment of J/Neo cells with CPT and CMEP-NQ resulted in 2.56 -fold and 1.32-fold increases in BAG3 and MCL-1 levels, respectively. Under these conditions, although the CPT-induced upregulation of the PUMA level was not markedly affected by CMEP-NQ, the CPT-induced caspase-9 activation and PARP cleavage were significantly inhibited by CMEP-NQ.

Consequently, our results indicate that the inhibitory action of CMEP-NQ against NOC-, 2-MeO-E_2_-, or CPT-induced BAK activation, which results in Δψm loss and caspase cascade activation, is attributable to CMEP-NQ-mediated upregulation of the anti-apoptotic BAG3 and MCL-1 proteins.

### Effect of CMEP-NQ on NOC-, 2-MeO-E_2_-, and CPT-induced cell cycle arrest in J/BCL-XL cells overexpressing BCL-XL

**Fig 4:**
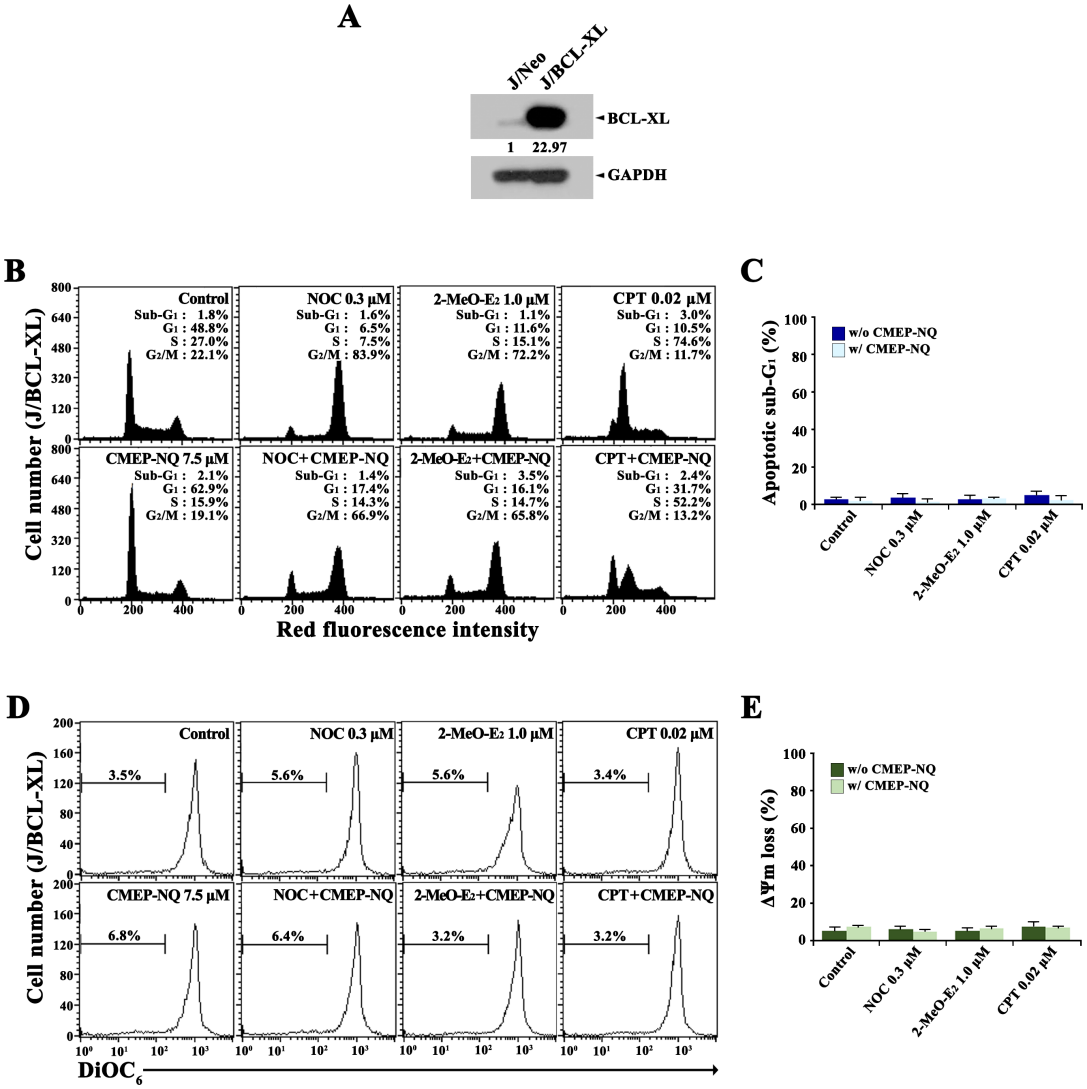
Western blot analysis of BCL-XL and GAPDH in J/Neo and J/BCL-XL cells (A), and flow cytometric analysis of the effect of CMEP-NQ on NOC-, 2-MeO-E_2_-, and CPT-induced alterations in cell cycle distribution (B and C) and Δψm loss (D and E) in Jurkat T cell clone transfected with a BCL-XL-expression vector (J/BCL-XL cells). After pretreatment with 7.5 μM CMEP-NQ for 1 h, J/BCL-XL cells (5 × 10^5^/ml) were treated with 0.3 μM NOC, 1.0 μM 2-MeO-E_2_, or 0.02 μM CPT for 17 h and then subjected to flow cytometric analysis of cell cycle distribution, Δψm loss, and BAK activation as described in the Materials and Methods. Western blot and flow cytometric analyses were performed as described in the Materials and Methods. A representative result is shown; two additional experiments yielded similar results. Error bars represent standard deviations.

The results of the flow cytometric analysis showed that the effect of CMEP-NQ on mitotic arrest caused by MDA (NOC- or 2-MeO-E_2_) treatment and S phase cell cycle arrest caused by CPT treatment was not significant in J/Neo cells. As treatment of J/Neo cells with NOC, 2-MeO-E_2_, or CPT resulted in 30–42% of apoptotic sub-G_1_ accumulation along with most of the remnant cells being arrested in the G_2_/M or S phase, it was likely that the effect of CMEP-NQ, if any, on the MDA- or DDA-induced cell cycle arrest would be more apparent in Jurkat T cells overexpressing anti-apoptotic BCL-2 or BCL-XL. In this context, we investigated the effect of CMEP-NQ on NOC-, 2-MeO-E_2_-, or CPT-induced apoptotic changes of cell cycle distribution in J/BCL-XL cells that appeared to express ~23-fold higher levels of BCL-XL compared to J/Neo cells (Fig 4A).

When J/BCL-XL cells were exposed to 0.3 μM NOC, 1.0 μM 2-MeO-E_2_, or 0.02 μM CPT for 16 h, the proportions of apoptotic sub-G_1_ cells in NOC-, 2-MeO-E_2_-, and CPT-treated cells were 1.6%, 1.1%, and 3.0%, respectively, showing that the overexpression of BCL-XL prevented the drug-treated cells from undergoing apoptosis (Fig 4B and 4C). Although the cells treated with NOC and 2-MeO-E_2_ were accumulated in the G_2_/M phase (83.9% and 72.2%, respectively), the number of G_2_/M cells decreased to 66.9% and 65.8%, respectively, and the number of G_1_ phase cells and S phase cells slightly increased in the presence of CMEP-NQ. Additionally, J/BCL-XL cells after CTP treatment included 10.5%, 74.6%, and 11.7% in G_1_, S, and G_2_/M phases, respectively, indicating that the majority of J/BCL-XL cells accumulated in the S phase. Concomitant treatment of J/BCL-XL cells with CPT and CMEP-NQ resulted in 31.7%, 52.2%, and 13.2% of cells in G_1_, S, and G_2_/M phases, respectively. Under these conditions, NOC-, 2-MeO-E_2_-, and CPT-induced mitochondrial damage, which led to Δ*ψ*m loss, was almost completely abrogated in JT/BCL-XL cells (Fig 4D and 4E).

These results confirm that the inhibitory effect of CMEP-NQ on MDA-induced mitotic or DDA-induced S phase cell cycle arrest was not remarkable in J/BCL-XL cells and that the protective action of CMEP-NQ against MDA- or DDA-induced mitochondrial damage and apoptosis was mediated by the inhibition of mitochondrial damage rather than by a disturbance in MDA-induced G_2_/M or DDA-induced S phase arrest.

### Effect of CMEP-NQ on NOC-, 2-MeO-E_2_-, and CPT-induced ROS production in J/Neo and J/BCL-XL cells

As excessive ROS generation in the cells is critical for the induction of apoptotic cell death [36–38], and as the mitochondrial apoptosis pathway frequently involves cytochrome *c* release and ROS generation [24,39], we examined whether the protective effect of CMEP-NQ on MDA- or DDA-induced apoptosis was mediated through the inhibition of drug-induced ROS generation using flow cytometric analysis with DHE staining. Additionally, to determine whether the inhibitory effect of CMEP-NQ on MDA- or DDA-induced ROS generation was associated with the prevention of mitochondrial damage that leads to Δψm loss and ROS generation, we compared the effect of CMEP-NQ on the induced ROS generation in J/Neo cells to that on the induced ROS production in J/BCL-XL cells.

**Fig 5:**
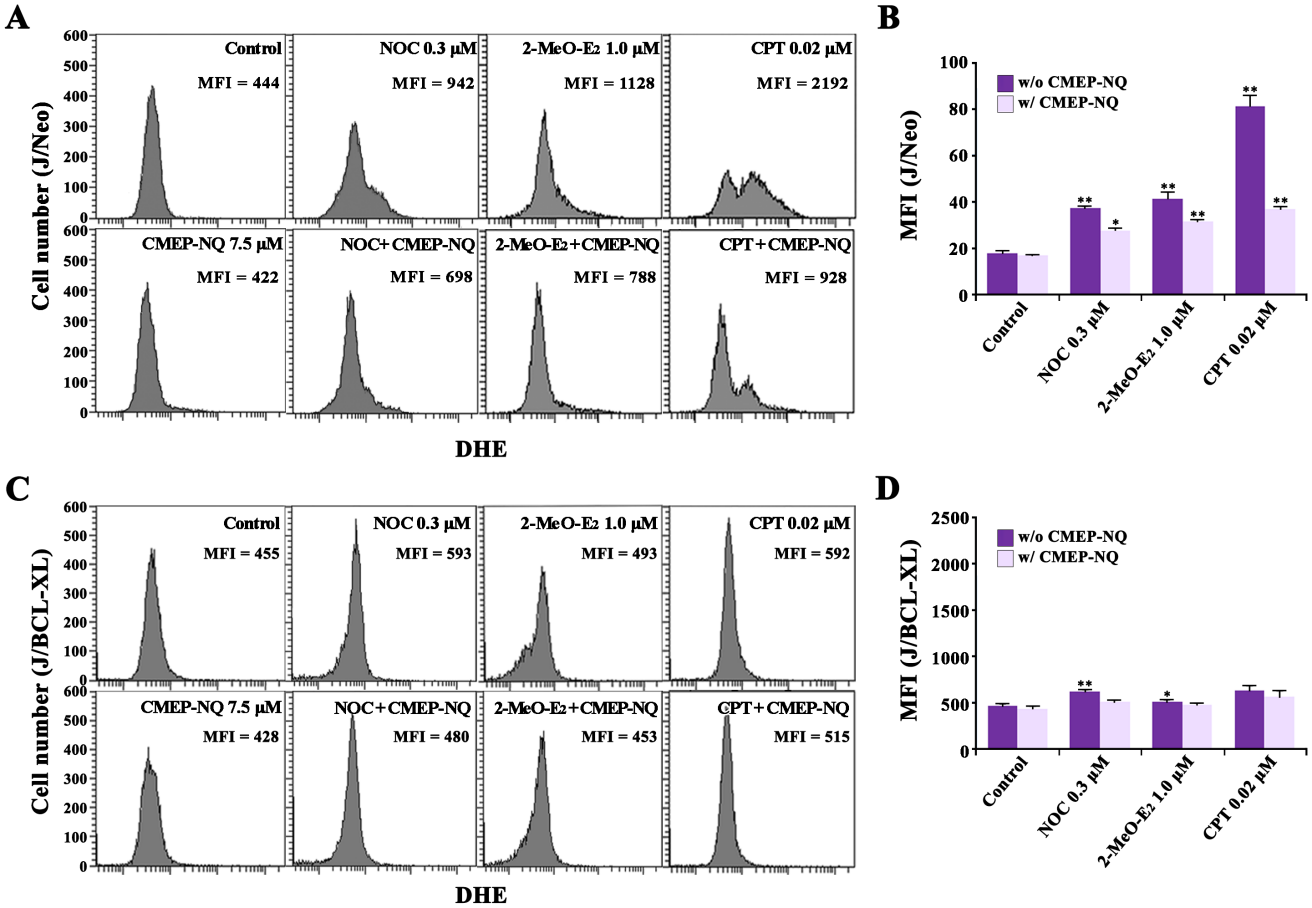
Flow cytometric analysis of ROS in J/Neo (A and B) and J/BCL-XL cells (C and D) following treatment with 0.3 μM NOC, 1.0 μM 2-MeO-E_2_, or 0.02 μM CPT. The cells (5 × 10^5^ ml/mL) were treated with the indicated concentrations of NOC, 2-MeO-E_2_ or CPT for 17 h. The intracellular ROS level was measured by flow cytometry and indicated by the mean fluorescent intensity (MFI) of the cells. A representative result is shown; two additional experiments yielded similar results. Error bars represent standard deviations with * and ** indicating *P <* 0.05 and *P <* 0.01, respectively, compared with the control.

Although the MFI value of exponentially growing J/Neo cells (the control) was 444, it increased by 2.1-, 2.5-, and 4.9-fold in NOC-, 2-MeO-E_2_-, and CPT-treated J/Neo cells, respectively (Fig 5A and 5B). At the same time, the MFI values of J/Neo cells concomitantly treated with each drug and CMEP-NQ increased only 1.6-, 1.8-, and 2.1-fold, respectively. These results demonstrate that NOC, 2-MeO-E_2_, and CPT elevated the intracellular ROS levels; however, the presence of CMEP-NQ resulted in a significant decrease in the drug-induced ROS production.

In contrast, J/BCL-XL cells showed only 1.3-, 1.1-, and 1.3-fold increases in MFI values after treatment with NOC, 2-MeO-E_2_, and CPT, respectively, indicating that the drug-induced ROS production in Jurkat T cells was inhibited by the overexpression of BCL-XL (Fig 5C and 5D). Under these conditions, however, none of the MFI values of J/BCL-XL cells observed following treatment with NOC, 2-MeO-E_2_, or CPT were further markedly reduced by concomitant treatment with CMEP-NQ.

**Fig 6:**
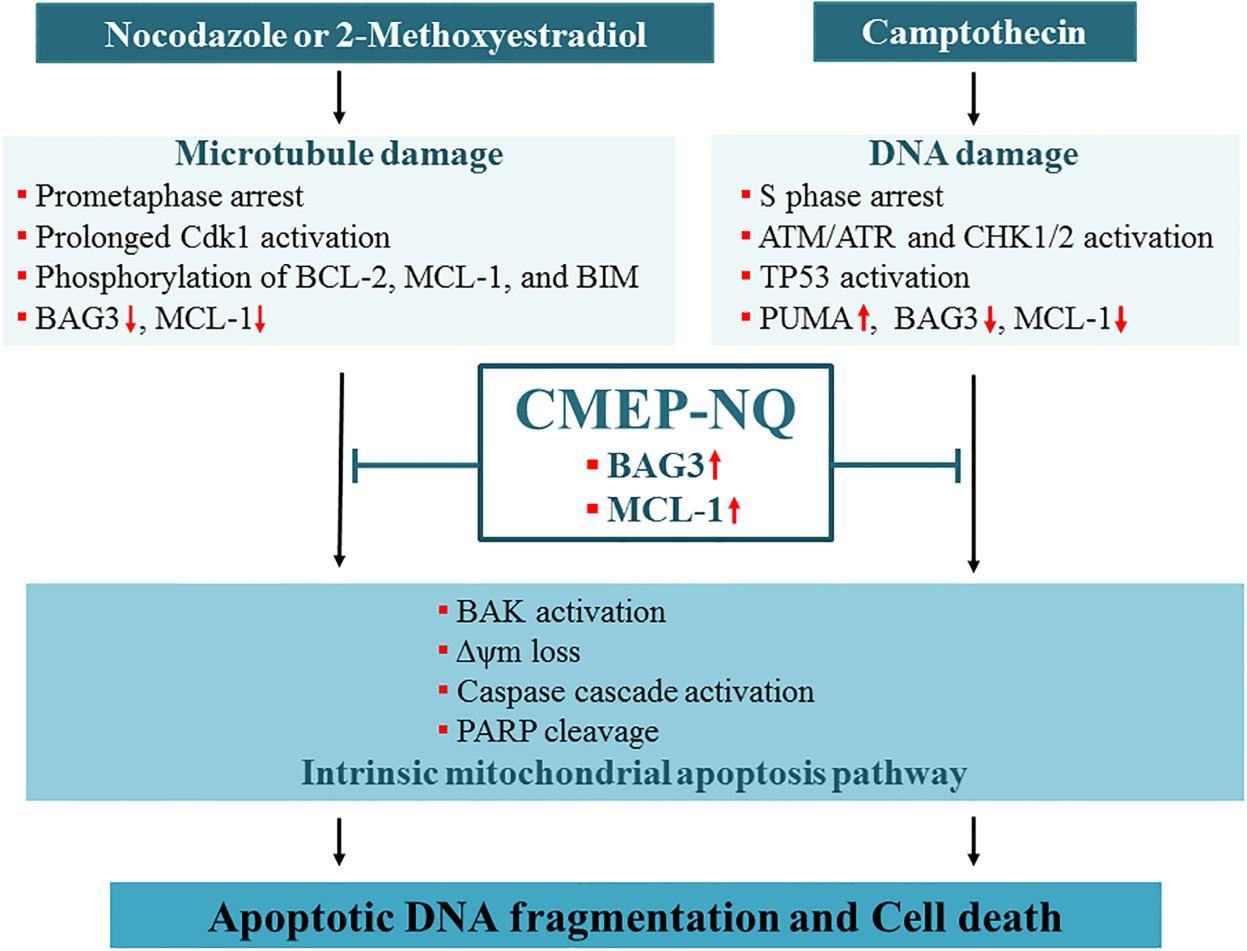
Schematic representation of the cytoprotective role of CMEP-NQ in MDA- or DDA-induced apoptotic cell death through elevation of anti-apoptotic BAG3 and MCL-1 levels.

Consequently, these results showed that NOC-, 2-Me-O-E_2_-, and CPT-induced ROS production that was attenuated by CMEP-NQ treatment occurred downstream of BCL-XL-preventable mitochondrial damage and thus downstream of CMEP-NQ-preventable BAK activation, causing mitochondrial damage, Δ*ψ*m loss, and caspase cascade activation (Fig 6). These results also confirm that CMEP-NQ-mediated abrogation of NOC-, 2-MeO-E_2_-, and CPT-induced apoptosis was attributable to a blockade of the BAK-dependent mitochondrial apoptotic pathway via the upregulation of anti-apoptotic BAG3 and MCL-1 levels rather than by direct inhibition of ROS generation.

## Discussion

In this study, we demonstrated for the first time that the CMEM-NQ-mediated cytoprotection against human Jurkat T cell apoptosis induced by MDAs (NOC and 2-MeO-E_2_) [32,33] or a DDA (CPT) [40] is mediated by blockade of BAK activation, leading to the prevention of mitochondrial damage and subsequent caspase cascade activation. Additionally, we showed that CMEP-NQ-mediated upregulation of the anti-apoptotic BAG3 and MCL-1 is associated with the blockade of induced BAK activation in NOC-, 2-MeO-E_2_-, and CPT-treated Jurkat T cells.

The anti-apoptotic BAG3 belongs to the group of six members of the BAG family (BAG1-6) that possess a highly conserved C-terminal BAG domain and contribute to multiple cellular processes, including cellular protein quality control, cell survival, and apoptosis [41,42]. The anti-apoptotic actions by which BAG3 protects cells from apoptotic cell death have been shown to be processed via stabilization of anti-apoptotic BCL-2 family members (BCL-2, BCL-XL, and MCL-1) in colon cancer RKO cells [43,44] as well as via direct interaction with pro-apoptotic BAX to attenuate its mitochondrial translocation in glioblastoma cells [45]. An additional anti-apoptotic action of BAG3 is mediated by its competition with BAG1 that positively cooperates with Hsp70 and CHIP (C-terminus of the Hsc70-interacting protein) to direct proteolytic substrates to the proteasome, resulting in the protection of IKK-γ from proteasome delivery and thus sustaining NF-kB activation and cell survival in osteosarcoma and melanoma cells [42]. Notably, it has also been reported that BAG3 positively regulates the expression level of MCL-1 through the down-regulation of miR-29b [46] and that the combined expression of BAG3 and MCL-1 confers resistance to chemotherapy-induced apoptosis in ovarian cancers [47]. Several studies have shown that the anti-apoptotic functions of BAG3 and MCL-1 can be suppressed by caspase-dependent cleavage to facilitate apoptotic cell death [48–50]. Furthermore, as a mechanism underlying the down-regulation of MCL-1 levels, MCL-1 phosphorylation at Ser-159/Thr-169 by CDK1, which leads to its destruction by the proteasome system, has been reported to be crucial for MDA-induced apoptosis [32,33,51,52].

Consistent with our previous results, Jurkat T cells treated with MDAs including NOC and 2-MeO-E_2_ commonly showed mitotic prometaphase arrest, CDK1 activation, phosphorylation of BCL-2, MCL-1, and BIM, and the onset of mitochondria-dependent apoptosis via triggering BAK activation, Δψm loss, and resultant caspase cascade activation [32,33,53]. Although CMEP-NQ was able to inhibit NOC- and 2-MeO-E_2_-induced BAK activation, Δψm loss, and caspase activation, it failed to inhibit NOC- and 2-MeO-E_2_-induced mitotic prometaphase arrest and CDK1 activation. This indicates that NOC- and 2-MeO-E_2_-induced apoptotic events upstream of BAK activation were not affected by the anti-apoptotic action of CMEP-NQ. On the other hand, exposure of Jurkat T cells to CPT, which is known to be a topoisomerase inhibitor that provokes DNA damage [54], resulted in apoptosis induction via the activating phosphorylation of ATM (Ser-1981), CHK1 (Ser-317), CHK2 (Ser-19), and p53 (Ser-15), BAK activation, Δψm loss, caspase activation, and PARP cleavage. CPT-induced BAK activation and its downstream events, such as caspase-9 activation and PARP cleavage, were significantly inhibited by CMEP-NQ, whereas the activating phosphorylation of ATM, CHK1, CHK2 and p53 and the upregulation of the pro-apoptotic PUMA level, as upstream events of BAK activation in the CPT-induced apoptotic signaling pathway [40], were not or were barely influenced by CMEP-NQ. Our results showed that the anti-apoptotic function of CMEP-NQ was exerted by the prevention of BAK activation, which was commonly required for MDA- and DDA-induced apoptotic cell death.

It is noteworthy that exposure of Jurkat T cells to CMEP-NQ alone resulted in not only a 2.34-fold increase in BAG3 levels but also a 1.35-fold increase in MCL-1 levels as evidenced by western blot analysis. Compared with the levels in untreated control cells, the intracellular levels of BAG3 and MCL-1 decreased significantly along with apoptosis induction in NOC-, 2-MeO-E_2_- and CPT-treated Jurkat T cells. However, the decrease in the BAG3 and MCL-1 levels in the individual drug-treated Jurkat T cells was commonly prevented by concomitant CMEP-NQ treatment. Under these conditions, the changes in the expression levels of the anti-apoptotic proteins BCL-2 and BCL-XL were negligible. The cytoprotective effect of CMEP-NQ, resulting from its anti-apoptotic function, was also observed in the proteasome inhibitor MG132-induced apoptosis of Jurkat T cells (data not shown), in which BAK-mediated mitochondrial damage and subsequent caspase cascade activation are critically involved [27]. In a recent study, we demonstrated that CMEP-NQ was able to inhibit TLR4-mediated proximal inflammatory responses such as sequential activation of the IRAK1/ TAK1/NF-κB pathway and ROS production without exerting cytotoxicity in LPS-stimulated RAW264.7 murine macrophage cells [20]. These previous and current results exclude the possible involvement of sustained NF-κB activation in the CMEP-NQ-mediated cytoprotection of cells from apoptosis. Consequently, the current results suggest that the protective action of CMEP-NQ, via upregulation of the anti-apoptotic BAG3 and MCL-1 proteins, against BAK-mediated mitochondrial apoptosis observed in the MDA (NOC or 2-MeO-E_2_)-treated Jurkat T cells, can be extended to DDA (CPT)- and proteasome inhibitor (MG132)-treated Jurkat T cells.

The excessive production of ROS has been reported to play an important role in apoptosis induction in human cells exposed to different apoptotic stimulants, including cisplatin, bleomycin, bortezomib, and UV irradiation [38,55]. In addition to the contribution of ROS to early stages of the mitochondrial apoptotic pathway to provoke mitochondrial dysfunction, an involvement of elevated ROS generation has been implicated in the execution of cell death after mitochondrial damage [33,56–58]. Previously, we showed that intracellular ROS levels which were markedly elevated in 2-MeO-E_2_-treated Jurkat T cells undergoing mitochondria-dependent apoptosis, are inhibited by the overexpression of BCL-2, indicating that ROS elevation occurred as a consequence of mitochondrial damage [33]. To examine whether preventing ROS generation is the proximal event responsible for the anti-apoptotic effect of CMEP-NQ on NOC-, 2-MeO-E_2_-, and CPT-induced apoptosis, the effect of CMEP-NQ on the individual drug-induced ROS production was compared in both J/Neo cells and J/BCL-XL cells overexpressing BCL-XL. As a result, not only 2-MeO-E_2_-induced ROS production but also NOC- and CPT-induced ROS generation was significantly reduced by BCL-XL overexpression, confirming that the drug-induced ROS production in Jurkat T cells occurred downstream of BAK-dependent mitochondrial damage, rather than as a proximal event causing BAK-dependent mitochondrial damage. In addition, although concomitant treatment with CMEP-NQ was able to significantly reduce the ROS production in NOC-, 2-MeO-E_2_-, and CPT-treated J/Neo cells, the reduced level appeared to be essentially similar to the reduced level by BCL-XL overexpression. As the BCL-XL protein is a well-known anti-apoptotic protein that protects cells from apoptotic cell death via the prevention of mitochondrial damage [25], our results are consistent with the following predictions: NOC-, 2-MeO-E_2_-, and CPT-induced ROS production occurs downstream of BAK-dependent mitochondrial damage, which can be targeted by anti-apoptotic BCL-XL, and CMEP-NQ-mediated reduction of ROS production is due to its inhibitory effect on BAK-dependent mitochondrial damage. Since BAK-mediated Δψm loss is one of the initial intracellular changes associated with mitochondria-dependent apoptosis [16], the above results suggest that CMEP-NQ may be a promising agent for mitochondria-targeted approaches that reduce unwanted mitochondrial damage and improve mitochondrial function.

## Conclusion

In this study, we have demonstrated that CMEP-NQ-mediated protection of Jurkat T cells from MDA- or DDA-induced apoptotic cell death is attributable to the elevation of anti-apoptotic BAG3 and MCL-1 levels, which leads to the inhibition of BAK conversion to its active form responsible for triggering the mitochondrial apoptosis pathway. Moreover, CMEP-NQ is able to block the mitochondrial damage-mediated intracellular ROS production in Jurkat T cells. These results are useful for evaluating the potency of CMEP-NQ as a promising agent for preventing mitochondrial dysfunction-associated cellular damage and diseases.
